# Supplementary material for: Mitochondrial fission factor (MFF) is a critical regulator of peroxisome maturation
Source: Biochim Biophys Acta Mol Cell Res. 2020 Jul;1867(7):118709. doi: 10.1016/j.bbamcr.2020.118709 (PMC7262603; doi:10.1016/j.bbamcr.2020.118709)
Supplement: Table S1 — Plasmids used in this study. [file mmc1.docx]

| Plasmid | Source |
| --- | --- |
| EGFP-SKL | Koch et al. 2005 |
| Myc-MFF | Gandre-Babbe and van der Bliek 2008 |
| c-roGFP2 | Ivashchenko et al. 2011 |
| mt-roGFP2 | Ivashchenko et al. 2011 |
| po-roGFP2 | Ivashchenko et al. 2011 |
| c-roGFP2-ORP1 | Lismont et al. 2019b |
| mt-roGFP2-ORP1 | Lismont et al. 2019b |
| po-roGFP2-ORP1 | Lismont et al. 2019b |
| pHRed-Cyto | Godinho and Schrader 2017 |
| pHRed-PO | Godinho and Schrader 2017 |
| HsPEX3(1-44)-EGFP | Fransen et al. 2001 |
