# Supplementary material for: Mitochondrial fission factor (MFF) is a critical regulator of peroxisome maturation
Source: Biochim Biophys Acta Mol Cell Res. 2020 Jul;1867(7):118709. doi: 10.1016/j.bbamcr.2020.118709 (PMC7262603; doi:10.1016/j.bbamcr.2020.118709)
Supplement: Table S2 — Primary and secondary antibodies used in this study. Abbreviations: IMF, immunofluorescence; WB, Western blot; pc, polyclonal; mc, monoclonal; ms, mouse; rb, rabbit; gt, goat; dk, donkey; HRP, horseradish peroxidase. [file mmc2.docx]

| Antibody | Type | Dilution  ______________  IMF WB | | Source |
| --- | --- | --- | --- | --- |
| ACBD5 | pc rb |  | 1:1000 | Proteintech (21080-1-AP) |
| ACOX1 | pc rb | - | 1:1000 | Proteintech (10957-1-AP)  or gift from T. Hashimoto, Japan |
| ATP synthase | mc ms | 1:500 | - | Abcam (ab14730) |
| α-Tubulin | mc ms | - | 1:1000 | Sigma (T9026) |
| Catalase | pc ms | 1:150 | - | Abcam (ab88650) |
| Catalase | mc rb | - | 1:250 | Abcam (ab179843) |
| GAPDH | pc rb | - | 1:5000 | ProSci (3783) |
| Myc | mc ms | 1:200 | - | Santa Cruz Biotechnology, Inc (9E10) |
| PEX5 | pc rb | - | 1:750 | Sigma (HPA039259) |
| PEX11β | mc rb |  | 1:1000 | Abcam (ab181066) |
| PEX14 | pc rb | 1:1400 | 1:4000 | D. Crane, Griffith University, Brisbane, Australia |
| PMP70 | pc rb | 1:100 | - | A. Völkl, University of Heidelberg, Heidelberg, Germany |
| PMP70 | mc ms | 1:500 | - | Sigma (SAB4200181) |
| Thiolase | pc rb | - | 1:2000 | Atlas antibodies (HPA007244) |
| Alexa Fluor 488 | dk anti-ms | 1:500 | - | ThermoFisher Scientific (A21202) |
| Alexa Fluor 488 | dk anti-rb | 1:500 | - | ThermoFisher Scientific (A21206) |
| Alexa Fluor 594 | dk anti-ms | 1:500 | - | ThermoFisher Scientific (A21203) |
| Alexa Fluor 594 | dk anti-rb | 1:500 | - | ThermoFisher Scientific (A21207) |
| HRP IgG | gt anti-ms | - | 1:10000 | Bio-Rad (170-6516) |
| HRP IgG | gt anti-rb | - | 1:10000 | Bio-Rad (172-1013) |
| IRDye 800 CW | gt anti-rb | - | 1:12500 | Westburg |
